# Mitragynine (Kratom)-Induced Cognitive Impairments in Mice Resemble Δ9-THC and Morphine Effects: Reversal by Cannabinoid CB_1_ Receptor Antagonism

**DOI:** 10.3389/fphar.2021.708055

**Published:** 2021-09-16

**Authors:** Ismail Nurul Iman, Nur Aimi Zawami Ahmad, Nurul Aiman Mohd Yusof, Ummi Nasrah Talib, Anwar Norazit, Jaya Kumar, Muhammad Zulfadli Mehat, Zurina Hassan, Christian P. Müller, Mustapha Muzaimi

**Affiliations:** ^1^ Department of Neurosciences, School of Medical Sciences, Universiti Sains Malaysia, Health Campus, Kota Bharu, Malaysia; ^2^ Department of Anatomy, School of Medical Sciences, Universiti Sains Malaysia, Health Campus, Kota Bharu, Malaysia; ^3^ Department of Biomedical Science, Faculty of Medicine, University of Malaya, Kuala Lumpur, Malaysia; ^4^ Department of Physiology, Universiti Kebangsaan Malaysia Medical Centre, Kuala Lumpur, Malaysia; ^5^ Department of Human Anatomy, Faculty of Medicine and Health Science, Universiti Putra Malaysia, Serdang, Malaysia; ^6^ Centre for Drug Research, Universiti Sains Malaysia, Minden, Malaysia; ^7^ Section of Addiction Medicine, Department of Psychiatry and Psychotherapy, University Clinic, Friedrich-Alexander-University of Erlangen-Nuremberg, Erlangen, Germany

**Keywords:** kratom, mitragynine, morphine, Δ9-tetrahydrocannabinol (THC), cannabinoid receptor 1 (CB1), cognition

## Abstract

Kratom is a widely abused plant-based drug preparation with a global interest in recent years, well beyond its native grounds in Southeast Asia. Mitragynine, its major psychoactive constituent is known to exhibit opioid-like behavioral effects with resultant neuroplasticity in the brain reward system. Its chronic administration is associated with cognitive impairments in animal studies. However, the underlying molecular mechanism for such a deficit remains elusive. In this study, the involvement of cannabinoid type-1 (CB_1_) receptors in cognitive deficits after chronic mitragynine exposures was investigated for 28 days (with incremental dose sensitization from 1 to 25 mg/kg) in adult male Swiss albino mice using the IntelliCage^®^ system. Chronic high-dose mitragynine exposure (5–25 mg/kg, intraperitoneal [i.p.]), but not low-dose exposure (1–4 mg/kg, i.p.), induced hyperlocomotion, potentiated the preference for sucrose reward, increased resistance to punishment, and impaired place learning and its reversal. Comparable deficits were also observed after chronic treatments with Δ-9-tetrahydrocannabinol (THC, 2 mg/kg, i.p.) or morphine (5 mg/kg, subcutaneous). Mitragynine-, morphine-, and THC-induced learning and memory deficits were reversed by co-treatment with the CB_1_ receptor antagonist, NIDA-41020 (10 mg/kg, i.p.). A significant upregulation of CB_1_ receptor expression was found in the hippocampal CA1 region and ventral tegmental area after chronic high-dose mitragynine and morphine, whereas a downregulation was observed after chronic THC. In conclusion, the present study suggests a plausible role of the CB_1_ receptor in mediating the dose-dependent cognitive deficits after chronic high-dose mitragynine exposure. This also highlights the potential of CB_1_ receptor antagonism in ameliorating the cognitive deficits associated with long-term kratom/mitragynine consumption in humans.

## Introduction

Kratom (*Mitragyna speciosa Korth*) is a native plant to the Philippine islands, New Guinea, and Southeast Asia, predominantly Malaysia, Thailand, and Indonesia. It is recognized as a local medicinal plant chiefly because of its antinociceptive and psychostimulant effects (Jansen and Prast, 1988; Hassan et al., 2013; Raffa, 2014). Since 2004, due to the growing concerns over the plant's narcotic properties and abuse liabilities, the Malaysian government has criminalized kratom's major alkaloid, mitragynine, under the Third Schedule of Poisons (Psychotropic Substances) Regulations, Poison Act 1952. Kratom is also currently regulated under the respective Narcotics Act in Thailand, Australia, and Myanmar (Bergen-Cico and MacClurg, 2016; Singh et al., 2017). Despite legal restrictions in such countries, the recreational use and abuse of kratom remains prevalent throughout Malaysia and Thailand (Ahmad and Aziz, 2012; Singh et al., 2017; Singh et al., 2019). In fact, claims and reports over the internet regarding its potential as a cheap opioid substitute have attracted the Western users to use kratom to self-medicate for opioid withdrawal and chronic pain, apart from being sold as a dietary supplement in recent decades in the United States and Europe (Boyer et al., 2008; Grundmann, 2017; Coe et al., 2019; Müller et al., 2020). The majority of long-term kratom users (over three-quarters) report developing dependence and an inability to cease its use, mainly due to its unpleasant withdrawal symptoms (Suwanlert, 1975; Boyer et al., 2008; Vicknasingam et al., 2010; Ahmad and Aziz, 2012; Saingam et al., 2013; Grundmann, 2017; Singh et al., 2019). Kratom and mitragynine are marketed for Western users either as a pure preparation (Cornara et al., 2013; Forrester, 2013; Coe et al., 2019) or as one herbal ingredient of “legal” or “herbal high” preparations, which are distributed in the form of powders, pills, and capsules under various names such as Krypton, K2, or Spice (Dresen et al., 2010; Arndt et al., 2011; Singh et al., 2014; Tavakoli et al., 2017). The emergence of reports on the serious adverse effects associated with kratom/mitragynine abuse has prompted a ban on kratom in several states in the United States (McWhirter and Morris, 2010; Nelsen et al., 2010; Holler et al., 2011; Kapp et al., 2011; Kronstrand et al., 2011; Forrester, 2013; Neerman et al., 2013; Trakulsrichai et al., 2013; Eggleston et al., 2019). Currently, the United States Drug and Enforcement Administration and Food and Drug Administration remain vigilant in considering to place kratom into Schedule I of the Controlled Substances Act (Henningfield et al., 2018).

Numerous studies on the major alkaloid, mitragynine, and the less abundant constituent, 7-hydroxymitragynine, of kratom have demonstrated the high binding affinity for supraspinal μ- and δ-opioid receptors governing the antinociceptive and antitussive actions of these constituents and their rewarding properties (Matsumoto et al., 1996; Thongpradichote et al., 1998; Takayama, 2004; Matsumoto et al., 2006; Yusoff et al., 2017). Several studies have demonstrated that acute and chronic administrations of mitragynine caused significant cognitive and emotional impairments in animals (Apryani et al., 2010; Hazim et al., 2011; Harizal et al., 2012; Yusoff et al., 2016; Iman et al., 2017; Hassan et al., 2019). Recent studies also reported that high doses of mitragynine (5 and 10 mg/kg) cause spatial/place learning deficit, accompanied by a disruption to synaptic transmission and long-term potentiation (LTP) at the CA1 field of the rat hippocampus, and electroencephalogram deficits (Hassan et al., 2019; Suhaimi et al., 2021). Thus far, the exact neural mechanisms that underlie these adverse effects on cognition remain elusive. In this context, little is known about low-dose mitragynine (1–4 mg/kg) despite the reported dose-dependent pharmacological effects of kratom/mitragynine—psychostimulants at low doses and opioid-like depressant effects at high doses (>5 mg/kg) (Suwanlert, 1975; Hassan et al., 2013; Saingam et al., 2013; Singh et al., 2019). Therefore, this study explores the low- and high-dose mitragynine range to reflect the spectrum of potential adverse effects on cognition and links the role of the endocannabinoid system for the first time to the best of the author's knowledge.

The endocannabinoid system and in particular the cannabinoid type-1 (CB_1_) receptors are well known for their role in the reinforcing effects of addictive substances and long-term behavioral sensitization after chronic use. This receptor system has been recognized for its reciprocal interaction with the opioid system and brain reward circuitry (Justinova et al., 2009; Katona, 2009; Scavone et al., 2013; Parsons and Hurd, 2015; Laredo et al., 2017; Manzanares et al., 2018). CB_1_ and opioid receptors co-localization at the brain areas governing motivation, learning and memory, and behavioral control lends support to this reciprocity (Pickel et al., 2004; Justinova et al., 2009; Wilson-Poe et al., 2012; Scavone et al., 2013; Manzanares et al., 2018). Changes in the expression level of CB_1_ receptors in response to drugs of abuse were observed in both animal and human studies, suggesting its contribution to long-term plasticity associated with drug administration (Justinova et al., 2009; Maldonado et al., 2013; Jin et al., 2014; Vlachou and Panagis, 2014; Manzanares et al., 2018). This study investigated the potential role of CB_1_ receptors in mediating the cognitive deficits induced by a chronic mitragynine sensitization.

## Materials and Methods

### Animals

A total of one hundred adult male Swiss albino mice (*n* = 100; weight: 30–35 g) were purchased from the breeding colony of the Animal Research and Service Centre of Universiti Sains Malaysia (Health Campus) in Kelantan, Malaysia. All mice were approximately 8–9 weeks old at the beginning of the experiment and naive. The mice were initially housed in groups of five per cage, for a minimum of 5 days before behavioral testing. These polypropylene cages had free access to standard commercial food pellets and tap water *ad libitum*. They were maintained under controlled environmental conditions (temperature, 22 ± 2°C; 50 ± 5% humidity; 12:12-h light/dark schedule). The physical behaviors of each mouse were observed throughout habituation and experimentation. Mice showing any signs of aggression (i.e., fighting/attacks and biting) that may affect their social behaviors were excluded from the analyses.

All the animals were maintained according to the specified duration of pre- and post-drug exposure. The experimental protocols for care and use of laboratory animals were approved by the Universiti Sains Malaysia (USM) Institutional Animal Care and Use Committee (USM IACUC) [Approval No: USM/AEA/2016/(101)(755)].

### Drugs and Chemicals

Mitragynine was supplied by the Centre for Drug Research, USM. Fresh *M. speciosa* leaves were harvested from Perlis, Malaysia, and authenticated by the Herbarium of the School of Biological Sciences, Universiti Sains Malaysia (Voucher No: USM1707-2017). Mitragynine, the active principal alkaloid of *M. speciosa* was isolated by the method reported by Beng et al. (2011) and Jamil et al. (2013). Purified mitragynine was confirmed by high-performance liquid chromatography and proton nuclear magnetic resonance (400 MHz) analyses (Jamil et al., 2013). Mitragynine obtained by this procedure was approximately 98% pure, with high stability at 4 to −20°C for 6 months. The dried mitragynine extract was sealed in a bottle and stored at 4°C until use with a prior inspection and written approval obtained from the Division of Pharmacy, Ministry of Health, Malaysia. Morphine sulfate (Pharmaniaga, Malaysia) and Δ-9-tetrahydrocannabinol [THC, Lipomed AG, Switzerland] were used as reference drugs. 20% Tween 20 (Bio-Rad Laboratories, United States) was used as a vehicle. NIDA-41020 (Sigma, United States) was used as the CB_1_ receptor antagonist.

### IntelliCage Apparatus

The IntelliCage^®^ system (TSE Systems GmbH, Bad Homburg, Germany) is a fully automated behavioral platform designed for short- and long-term cognitive monitoring of individual radio frequency–tagged (RFID) mice living in social groups, as described in detail in earlier studies (Galsworthy et al., 2005; Lipp et al., 2005; Endo et al., 2011; Kiryk et al., 2020). Briefly, the IntelliCage is a standard polycarbonate cage (55 cm width × 38 cm depth × 21 cm height), which accommodates up to 16 mice at a time. A triangular operant test chambers (15 × 15 × 21 cm) are fitted at each of the corners. Entry (or visit) into each operant chamber is identified by a circular RFID antenna that detects the animal's unique ID tags and records their visits. Two round apertures, equipped with motorized doors, on each chamber wall permit free access to water bottles. Small motorized doors at the apertures can be programmed to close to limit water access and are able to detect mouse nose poke patterns (individual pokes at each doors). Animals can be trained to perform fixed or progressive ratio nose pokes at the door to allow access to water. Mounted above each door is a motorized valve for delivery of air-puffs as a form of negative reinforcement or punishment.

### Behavioral Design in the IntelliCage System

All animals were subcutaneously (s.c.) implanted with sterile glass-covered microtransponders (12 × 2 mm; Datamars, Switzerland) for individual recognition in the IntelliCage using supplied disposable syringe and an injector (Datamars, Switzerland). After 48 h of implantation, all animals were checked for microtransponder retention with a handheld electronic reader (Datamars, Switzerland) before being released into the IntelliCage.

#### Drug Sensitization

The incentive-sensitization theory of addiction posits the progressive increase in the neurological and behavioral stimulatory effects of a drug following repeated intermittent administration. The drug-induced hypersensitization of the brain reward circuits is hypothesized to cause a pathological transition from drug “liking” to “wanting” that underlies compulsive substance use as previously demonstrated with chronic challenges of morphine, cocaine, ethanol, nicotine, and cannabinoids (Berridge and Robinson, 2011; Marinho et al., 2015; Grigutsch et al., 2019; Lopes et al., 2020).

All mice were taken out from the IntelliCage once daily (in the morning) to receive their assigned 28-day drug sensitization regimen. In the drug + NIDA-41020 groups, mice were sensitized with mitragynine, morphine, or THC for the first 14 days (Richards et al., 1999; Paine et al., 2003; Olmstead, 2006; Yusoff et al., 2016), whereas NIDA-41020 (without coadministration of mitragynine, morphine, or THC) was administered starting from Day 15 onward. The interventions for the receptor antagonist groups were designed to eliminate any mitragynine, morphine, or THC cross-interaction with opioid and/or other receptor systems, thus avoiding any interference with the study objectives. All drug sensitizations were performed at a volume of 1 ml/kg body weight. Mice were returned to IntelliCage immediately after injection handling. All mice were randomly assigned to 10 groups (*n* = 10/group). Treatment details are shown in Table 1.

**TABLE 1:** Description of experimental groups and drug sensitization.

	Intervention groups	Description
1	Untreated	The untreated group that served as a negative control
2	Vehicle control	Daily i.p. injection of 20% Tween-20 (1 ml/kg) for 28 days
3	Morphine	Daily s.c. injection of morphine sulfate (5 mg/kg) for 28 days
4	Morphine + NIDA-41020	Daily s.c. injection of morphine sulfate (5 mg/kg) from Day 1 to Day 14, followed by daily i.p. administration of NIDA-41020 (10 mg/kg) from Day 15 to Day 28
5	THC	Daily i.p. injection of THC (2 mg/kg) for 28 days
6	THC + NIDA-41020	Daily i.p. injection of THC (2 mg/kg) from Day 1 to Day 14, followed by daily i.p. administration of NIDA-41020 (10 mg/kg) from Day 15 to Day 28
7	Mit high	Daily i.p. injection of mitragynine (from 5 to 25 mg/kg; in increments of 5 mg/kg) for 28 days
		Day 1–3 = 5 mg/kg of mitragynine
		Day 4–6 = 10 mg/kg of mitragynine
		Day 7–9 = 15 mg/kg of mitragynine
		Day 10–12 = 20 mg/kg of mitragynine
		Day 13–28 = 25 mg/kg of mitragynine
8	Mit high + NIDA-41020	Daily i.p. injection of mitragynine (from 5 to 25 mg/kg; in increments of 5 mg/kg) from Day 1 to Day 14, followed by daily i.p. administration of NIDA-41020 (10 mg/kg) from Day 15 to Day 28
		Day 1–3 = 5 mg/kg of mitragynine
		Day 4–6 = 10 mg/kg of mitragynine
		Day 7–9 = 15 mg/kg of mitragynine
		Day 10–12 = 20 mg/kg of mitragynine
		Day 13–14 = 25 mg/kg of mitragynine
		Day 15–28 = 10 mg/kg of NIDA-41020
9	Mit low	Daily i.p. injection of mitragynine (from 1 to 4 mg/kg; in increments of 1 mg/kg) for 28 days
		Day 1–3 = 1 mg/kg of mitragynine
		Day 4–6 = 2 mg/kg of mitragynine
		Day 7–9 = 3 mg/kg of mitragynine
		Day 10–28 = 4 mg/kg of mitragynine
10	Mit low + NIDA-41020	Daily i.p. injection of mitragynine (from 1 to 4 mg/kg; in increments of 1 mg/kg) from Day 1 to Day 14, followed by daily i.p. administration of NIDA-41020 (10 mg/kg) from Day 15 to Day 28
		Day 1–3 = 1 mg/kg of mitragynine
		Day 4–6 = 2 mg/kg of mitragynine
		Day 7–9 = 3 mg/kg of mitragynine
		Day 10–14 = 4 mg/kg of mitragynine
		Day 15–28 = 10 mg/kg of NIDA-41020

NIDA-41020, Sigma, United States, is the CB_1_ receptor antagonist.

i.p., intraperitoneal; Mit high, high-dose mitragynine; Mit low, low-dose mitragynine; s.c., subcutaneous; THC, Δ-9-tetrahydrocannabinol.

The incremental dosage of mitragynine (low and high) were selected to mimic human kratom consumption, which often develops into dependency and tolerance (i.e., increase number of kratom leaves and frequency of intake) after prolonged consumption (Saingam et al., 2013; Singh et al., 2014; Singh et al., 2019). The selected low-dose mitragynine (from 1 to 4 mg/kg; in increments of 1 mg/kg) has been shown to produce light stimulant effects (Yusoff et al., 2016). The selected dose and route of high-dose mitragynine administration (from 5 to 25 mg/kg; in increments of 5 mg/kg) have previously been shown to affect locomotion, cognition, and memory functions in mice (Apryani et al., 2010; Yusoff et al., 2016; Iman et al., 2017; Hassan et al., 2019). The selected doses and routes of administration of morphine sulfate (5 mg/kg, s.c.) and THC (2 mg/kg, intraperitoneal [i.p.]) have been shown to develop profound morphine- (Abdel-Zaher et al., 2013) and THC-induced (Vlachou et al., 2007; Zuardi et al., 2012; Iman et al., 2017) tolerance and dependence in mice, respectively, without any confounding toxic effects. The dose of CB_1_ receptor antagonist NIDA-41020 (10 mg/kg, i.p.) was chosen because it effectively attenuated behavioral effects of morphine in a previous study (Bdeer et al., 2014).

#### Behavioral Parameters in the IntelliCage System

**FIGURE 1:**
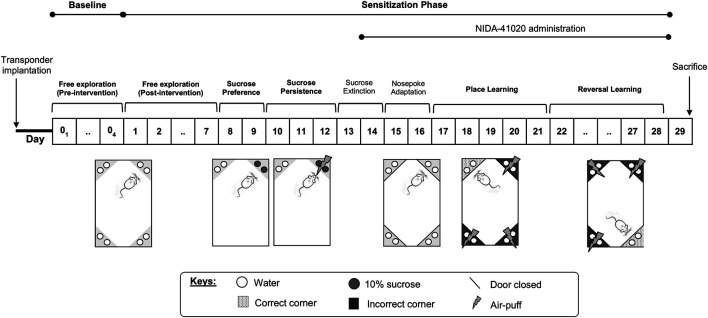
Summary of the IntelliCage experimental protocol.

Following their introduction to the IntelliCage, the mice were allowed free access to all IntelliCage corners for 4 days to measure their baseline exploratory behaviors before drug intervention (see Baseline Phase section). Subsequently, the mice were subjected to daily sensitization for 28 days (see Sensitization Phase section) according to their assigned drug intervention. The following parameters (Figure 1) have been adapted with modifications from Radwanska and Kaczmarek (2012), as also described previously (Iman et al., 2017), and applied through appropriately designed learning protocols in the IntelliCage software as has been given below.

##### Baseline Phase

During the pre-intervention days, mice were given free access to all cage areas for 4 days without receiving any drug intervention. All water-access doors were opened. Animals were tested for exploratory activity in the novel cage/environment, measured as the number of visits during the first hour in the IntelliCage, and in the familiar cage/environment, measured as the number of visits per day during the following 3 consecutive days.

##### Sensitization Phase

Activity in the familiar environment (post-intervention, Days 1–7): the IntelliCage setup was identical to the baseline phase. Data collected comprised of the number of visits per day for 3 consecutive days following drug sensitization (Day 5–7). As established in previous studies, the mean number of corner visits was used as a proxy for general exploratory activities (Galsworthy et al., 2005; Radwanska and Kaczmarek, 2012) for comparison with baseline exploratory activities.

Sucrose reward preference (Day 8 and 9): mice had access to normal tap water at one active corner and 10% sucrose solution at the other active corner. The doors of the two remaining inactive corners were closed throughout this protocol.

Persistence in sucrose-seeking (Day 10–12): mice were subjected to air-puff punishment (0.4 bar, 2-s duration) following sucrose-reward drinking. The doors of the two remaining inactive corners were closed throughout this protocol.

Sucrose extinction (Day 13 and 14): the IntelliCage setup identical to the baseline phase was used to prepare the mice for the subsequent protocol.

Nose poke adaptation (Day 15 and 16): water-access doors were closed at all corners. Mice had to perform one nose poke, which opened the respective door, in order to drink. Opened doors were automatically closed after 5 s. The least preferred corners of the individual mouse were determined for programming the subsequent place learning protocol.

Place learning (Day 17–21): all doors remained closed, but access to water was restricted to one water-rewarded corner (the individual least preferred corner during nose poke adaptation) termed the “correct corner.” Three successive nose pokes at the individual correct corner opened the respective door for 5 s. The percentage of visits with nose pokes to the correct corner were determined as an indicator for learning.

Reversal learning (Day 22–28): the same procedure as for place learning was followed, but the correct water-rewarded corner was reversed to the diagonal opposite corner. The percentage of visits with nose pokes in the newly placed correct corner was measured as an indicator for reversal learning.

### Brain Sample Collection

Following 24 h after the end of the IntelliCage study (Day 29), all mice were euthanized with pentobarbital (10 mg/kg, i.p.). Mice for an immunohistochemistry study were transcardially perfused with phosphate-buffered saline, followed by 10% (v/v) neutral buffered formalin (NBF), with a steady flow rate of 10.0 ml/min using an IPC Digital Peristaltic Pump (Ismatec, Germany). The whole brain of each mouse was isolated and cut along the hemispheric fissure with a sharp blade, dividing the brain into halves, and post-fixed in 10% (v/v) NBF at room temperature overnight before tissue processing. Mice for the western blot and quantitative real-time polymerase chain reaction (qPCR) studies were decapitated under pentobarbital euthanasia, followed by rapid removal of their brain on ice and dividing it into the two hemispheres. The cerebral hemisphere tissues collected for the western blot study were immediately stored at −80°C, while the tissues for the qPCR study were immersed in RNAlater^®^ solution and stored at −80°C until further use.

### Immunohistochemistry

Immunohistochemistry staining was performed to investigate CB_1_ receptor expression in the mouse hippocampus and ventral tegmental area (VTA) brain regions after chronic drug treatment. For assessment of the specificity of the reaction, positive controls (mouse cerebellum) (Ashton et al., 2004) and negative controls (incubation without primary antibodies) were routinely included.

The processed mice brain tissues blocked in paraffin were cut into a series of 4-μm-thick sagittal sections using a microtome. The hippocampus and VTA brain regions were determined using the Allen Mouse Brain Atlas^©^ online portal (Allen Institute for Brain Science, 2004) and visualized under a light microscope. Systemic random sampling technique was used to select one in every five sections for a total of four sections per brain. The selected ribbons of sections were mounted on poly-L-lysine–coated slides, air-dried overnight, deparaffinized with xylene, cleared, and rehydrated in graded ethanol.

Following reduction of endogenous peroxides through pre-incubation with 1% hydrogen peroxide (Merck, Germany), the sections were microwaved for antigen retrieval in 1X Tris-EDTA (pH 9.0) for 20 min. The sections were then blocked for nonspecific background staining with 5% BSA (Sigma, United States; 15 min RT) and incubated overnight at 4°C with rabbit polyclonal primary antibody against cannabinoid receptor type-1 (Anti-CB_1_ receptor; Cat No: ab23703; Abcam, United Kingdom; dilution 1:200). Sections were then incubated with horseradish peroxidase (HRP)–conjugated secondary antibody (goat anti-rabbit IgG H+L HRP; Cat No: ab205718; Abcam, United Kingdom; dilution 1:1,000; 1 h RT). Sections were stained using the 3,3′-diaminobenzidine (DAB) Enhanced Liquid Substrate System (Sigma, United States) for chromogenic detection and counterstained with hematoxylin. Each step was followed by an appropriate wash per triplicate in Tris-buffered saline and 0.5% Tween-20 (Bio-Rad, United States. The sections were then dehydrated in ascending concentrations of ethanol, cleared in xylene, and mounted. The staining pattern was assessed using a light microscope according to the DAB chromogen reaction uptake.

Digital images of immunohistochemical staining were observed and captured using an Olympus BX41 microscope with Olympus cellSens Standard software (Version 1.16; Tokyo, Japan). CB_1_ receptor immunoreactivity was quantified from the optical density (OD) of DAB signal using color deconvolution paradigm in NIH Image J software (Ruifrok and Johnston, 2001). The systematic random sampling technique was used to select the area of interest of the CA1 hippocampal and VTA regions for OD analysis. The measured OD was automatically corrected against the white background value. The immunoreactive density profile from at least 15 sections per group was then averaged to determine the mean OD value. A histogram of the OD values was plotted for further statistical analyses.

### Western Blot

The frozen brain tissues were homogenized using syringe-based technique in 20 volumes of T-PER™ Tissue Protein Extraction Reagent and Halt™ Protease and Phosphatase Inhibitor Cocktail (Thermo Scientific, United States). Brain homogenates were centrifuged at 10,000 × *g* for 10 min at 4°C. The supernatant was aspirated, and the protein lysate obtained was kept at −20°C until further analysis. All procedures were performed using prechilled reagents on ice. Total protein concentrations were determined using Quick Start™ Bradford Protein Assay kit (Bio-Rad, United States) and NanoDrop™ 2000/2000c Spectrophotometer (Thermo Scientific, United States).

Protein lysates (30 μg) were individually heated at 95°C for 10 min in 2X Laemmli sample buffer (1:1 ratio) and resolved by electrophoresis in 10% sodium dodecyl sulfate (SDS)–polyacrylamide gel using Mini-PROTEAN^®^ electrophoresis tank (Bio-Rad, United States) at 100 V for 90 min in 1X Running Buffer (Tris-glycine SDS, pH 8.3, Bio-Rad, United States). The gel was transferred onto a polyvinylidene fluoride (PVDF) membrane using the iBlot™ 2 Dry Blotting System using preprogramed voltage combinations (i.e., 20 V for 1 min, 23 V for 4 min, and 25 V for 2 min). Blotted PVDF membranes were incubated in 10 ml 1X iBind™ Solution for 10 min at RT to block nonspecific binding. The blocked membranes were then assembled onto the iBind Western System and sequentially incubated with anti–CB_1_ receptor primary antibody (dilution 1:1,000) and HRP-conjugated secondary antibody (dilution 1:2000).

Immunoreactive protein bands were visualized using Pierce™ ECL Western Blotting Substrate (Thermo Scientific, United States) and placed onto the Fusion^©^ FX7 Molecular Imager platform (Vilber Lourmat, France) for resulting signal image acquisition. Anti-β-actin antibody (dilution 1:2000) was used as a loading control for the western blot study. The CB_1_ receptor immunoreactive band intensities were quantified using Image J software (NIH Image, United States) and normalized to β-actin control. A band normalization–arbitrary value histogram was plotted for further statistical analysis.

### Quantitative Real-Time PCR

Total RNAs from brain tissue homogenates were extracted using GeneJET™ RNA Purification Kit (Thermo Scientific, United States) following the manufacturer's instructions. Extracted RNA samples were measured for total RNA concentration and purity using NanoDrop™ 2000/2000c Spectrophotometer and checked for RNA integrity using 1% agarose gel electrophoresis. Subsequently, 500 ng of the total RNA was converted to complementary DNA (cDNA) in 20-µl reactions using AffinityScript QPCR cDNA Synthesis Kit (Agilent, United States), consisting of 2X First-Strand Master Mix, AffinityScript RT/RNase Block Enzyme Mixture, 0.1 µg/µl Oligo (dT) and random primers, following the manufacturer's instructions. Each cDNA template reaction was then adjusted to 50 µl using RNase-free water and kept at −20°C until use.

Each qPCR assay was prepared with 50-ng cDNA template in a 20-µl reaction in triplicate using 2X Brilliant III Ultra-Fast QPCR Master Mix with ROX reference dye (Agilent, United States) on Stratagene Mx3005P Real-time PCR Machine (Agilent, United States). The primer-probe sets used were predesigned PrimeTime qPCR Assays (Integrated DNA Technologies, United States), as given below:

*Cnr1* (GenBank^®^ Accession No. NM_007726; 25 bp):
   GCA​AAT​TTC​CTT​GTA​GCA​GAG​AG (forward),
   TGA​GAA​AGA​GGT​GCC​AGG​A (reverse) and
   /56FAM/ACAGGTGCC/ZEN/GAGGGAGCTTC/3IABkFQ/(probe);
 β*-actin* housekeeping gene (GenBank Accession No. NM_007393; 25 bp):
   GAT​TAC​TGC​TCT​GGC​TCC​TAG (forward),
   GAC​TCA​TCG​TAC​TCC​TGC​TTG (reverse) and
   /56-FAM/CTGGCCTCA/ZEN/CTGTCCACCTTCC/3IABkFQ/(probe).

Each qPCR assay was validated using 5-point serial dilutions of the first-strand cDNA template with PCR efficiency rates between 96 and 102% with *R* ^2^ > 0.990. The thermal cycling incubation conditions for qPCR analysis were activation at 95°C for 3 min, denaturation at 95°C for 15 s, and annealing at 55°C for 20 s for 40 cycles. Relative mRNA expression of the *Cnr1* gene was determined using Relative Expression Software Tool (REST^©^) software and normalized to the β-actin reference gene. Subsequently, gene normalization–expression ratio histograms were plotted for further statistical analysis.

### Statistical Analyses

Results were analyzed as mean ± standard error of mean (SEM). In the IntelliCage study, comparison between groups was performed using one-way or two-way repeated measures of ANOVA, followed by *post-hoc* Tukey test. One-way ANOVA with *post-hoc* Tukey test was used to analyze the CB_1_ receptor expression in immunohistochemistry and the western blot studies, as well as *Cnr1* gene expression in the qPCR study. All statistical procedures were performed using GraphPad Prism version 8.0. For all analyses, *p* < 0.05 was accepted to be statistically significant.

## Results

### Mitragynine Enhances Exploratory Activity in the IntelliCage

**FIGURE 2:**
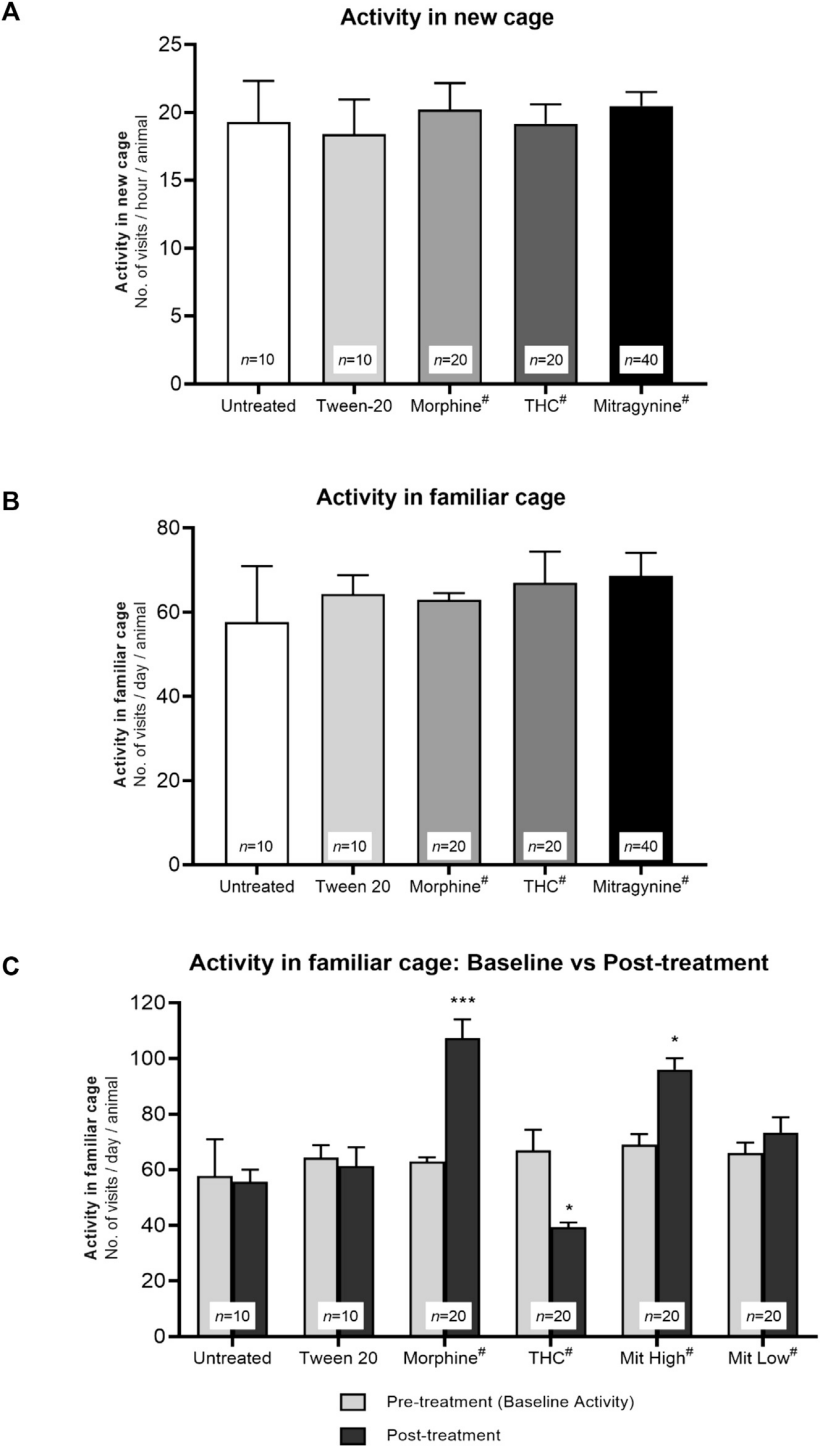
Effects of drug intervention on exploratory activities. Baseline exploratory activity in **(A)** novel and **(B)** familiar environment. The mean number of visits to all corners per test group during the first hour in the novel IntelliCage and 3 consecutive days in the familiar IntelliCage environment throughout the baseline phase. Without any drug intervention, all groups in **(A)** and **(B)** showed no significant differences in novelty-induced exploration and baseline daily activity (*p* > 0.05; one-way ANOVA). **(C)** Effects of drug sensitization to daily activity in the familiar IntelliCage environment. The mean number of visits to all corners during 3 days of baseline phase vs 3 days of sensitization phase (Day 5–7). Morphine and high-dose mitragynine–treated mice showed a significant increment in their general familiar vs baseline activities, in which the increments are comparable (not significant) between mitragynine vs morphine. By contrast, THC-treated mice showed a significant decrease in general activity. *p < 0.05 vs baseline; **p < 0.01 vs baseline (two-way repeated measures ANOVA followed by *post-hoc* Tukey test). ^#^includes animals from drug only and drug + receptor antagonist groups (i.e., each group *n* = 10).

Novelty-induced exploration of mice was measured by the mean number of visits to all corners during the first hours in the novel IntelliCage, while daily exploratory behaviors were measured by the mean number of visits to all corners during three consecutive days in familiar IntelliCage environment. No mice received any drug and/or receptor antagonist intervention during the course of the baseline exploratory phase. Therefore, as expected from the predrug baseline phase, no significant difference between the groups was displayed with regard to the number of corner visits performed in the novel environment (Figure 2A; F_4, 95_ = 0.883, *p* = 0.919) or familiar environment (Figure 2B; F_4, 95_ = 1.161, *p* = 0.859).

The number of visits post–drug sensitization was measured by the mean number of daily corner visits during Day 5–7 of sensitization, as compared with baseline visits. A two-way repeated measures ANOVA found statistically significant difference in exploratory activities between the baseline and sensitization phases across groups (Figure 2C; effect of treatment: F_5, 94_ = 7.96, *p* = 0.0016; effect of day: F_1, 94_ = 6.03, *p* = 0.032; treatment × day interaction: F_5, 94_ = 10.86, *p* = 0.0004). The morphine and high-dose mitragynine (5–25 mg/kg) groups performed significantly more corner visits following drug sensitization than the baseline group (morphine: *p* < 0.001, high-dose mitragynine: *p* = 0.015), with no significant difference between both groups (mitragynine vs morphine: *p* > 0.5804). By contrast, THC-sensitized mice performed significantly fewer corner visits than their baseline visits (*p* = 0.011). The untreated, Tween 20 vehicle, and low-dose mitragynine (1–4 mg/kg) groups did not differ significantly between their baseline and post-intervention exploratory activities in the familiar IntelliCage environment (untreated: *p =* 0.9998; Tween 20: *p* = 0.9983; low-dose mitragynine: *p* = 0.929).

### Mitragynine Enhances Sucrose Reward Preference in the IntelliCage

**FIGURE 3:**
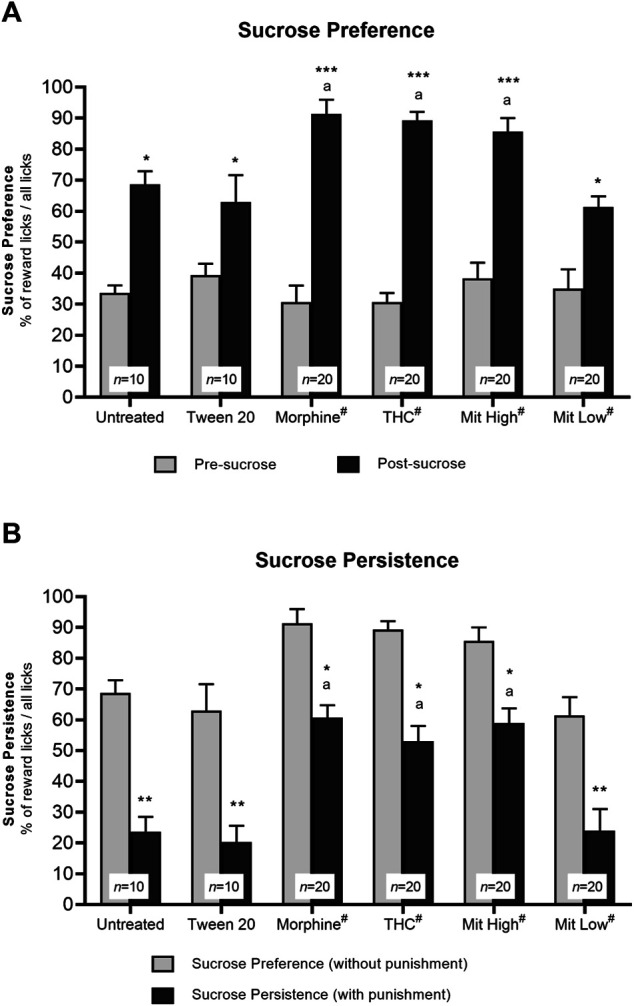
Effects of drug intervention on preference for natural reward (sucrose) and persistence for sucrose seeking. **(A)** Preference for sucrose reward. The percentage of licks at the natural reward (10% sucrose) corner during the 2-day sucrose phase vs pre-sucrose (*p < 0.05, **p < 0.01, ***p < 0.001 vs pre-sucrose; a: *p* < 0.05 vs Tween 20; ns = *p* > 0.05 vs morphine; two-way repeated measures ANOVA with *post-hoc* Tukey test). **(B)** Persistence of sucrose seeking. The percentage of reward licks when paired with air-puff punishment measured during the 3-day phase vs without air-puffs. *p < 0.05; **p < 0.01 vs reward licks without punishment; a: *p* < 0.05 vs Tween 20 (two-way repeated measures ANOVA with *post-hoc* Tukey). ^#^includes animals from drug only and drug + receptor antagonist groups (i.e., each group *n* = 10).

In the sucrose preference test, all mice showed strong preference for sucrose over water as demonstrated by the overall marked increase in lick preference for the corner once it was associated with sucrose as opposed to pre-sucrose (Figure 3A; effect of time: F_1, 94_ = 259.3, *p* < 0.001; effect of treatment: F_5, 94_ = 2.925, *p* = 0.403; treatment × time interaction: F_5, 94_ = 6.315, *p* = 0.004; in untreated: *p* = 0.008; Tween-20: *p* = 0.018; morphine: *p* < 0.001; THC: *p* < 0.001; high-dose mitragynine: *p* < 0.001; low-dose mitragynine: *p* = 0.0084 vs pre-sucrose). Morphine-, THC-, and high-dose mitragynine-sensitized groups elicited stronger preference for sucrose reward than untreated and vehicle control groups (morphine group: *p* = 0.0214; THC group: *p* = 0.0340; mitragynine group: *p* = 0.0463 vs untreated). Interestingly, the mice in the high-dose mitragynine group showed a similar preference for sucrose reward as those in the morphine and THC groups (*p* = 0.9857 vs morphine, *p* = 0.9979 vs THC), which may suggest comparable reward-seeking traits associated with prolonged high-dose mitragynine administration. The low-dose mitragynine group, however, showed comparable sucrose preference as the control group (*p* = 0.9963 vs untreated).

### Mitragynine Enhances Resistance to Punishment in Sucrose Seeking in the IntelliCage

Two-way repeated measures ANOVA showed a statistically significant reduction in sucrose consumption in all mice, irrespective of the intervention groups, following air-puff punishment (Figure 3B; effect of time: F_1, 94_ = 118.3, *p* < 0.001; treatment × time interaction: F_5, 94_ = 6.315, *p* = 0.004), with statistically significance effect observed between groups (F_5, 94_ = 40.27, *p* < 0.0001). *Post-hoc* comparisons indicated that sensitized mice were significantly more resistant to punishment when reward licks were associated with air-puff punishment (morphine: *p* = 0.0226; THC: *p* = 0.0177; high-dose mitragynine: *p* = 0.0495 vs reward licks without air-puff punishment) as compared with those in the control groups (untreated: *p* = 0.0016; Tween-20: *p* = 0.0025). Furthermore, all three sensitized groups showed a similar ability to withstand and resist air-puff punishment to obtain sucrose reward (high-dose mitragynine: *p* = 0.9968 vs morphine; THC: *p* = 0.9880 vs morphine). Meanwhile, the low-dose mitragynine group showed comparable resistance to punishment as the control group (*p* = 0.9996 vs untreated).

### Mitragynine Impairs Place Learning and Reversal Learning in the IntelliCage

**FIGURE 4:**
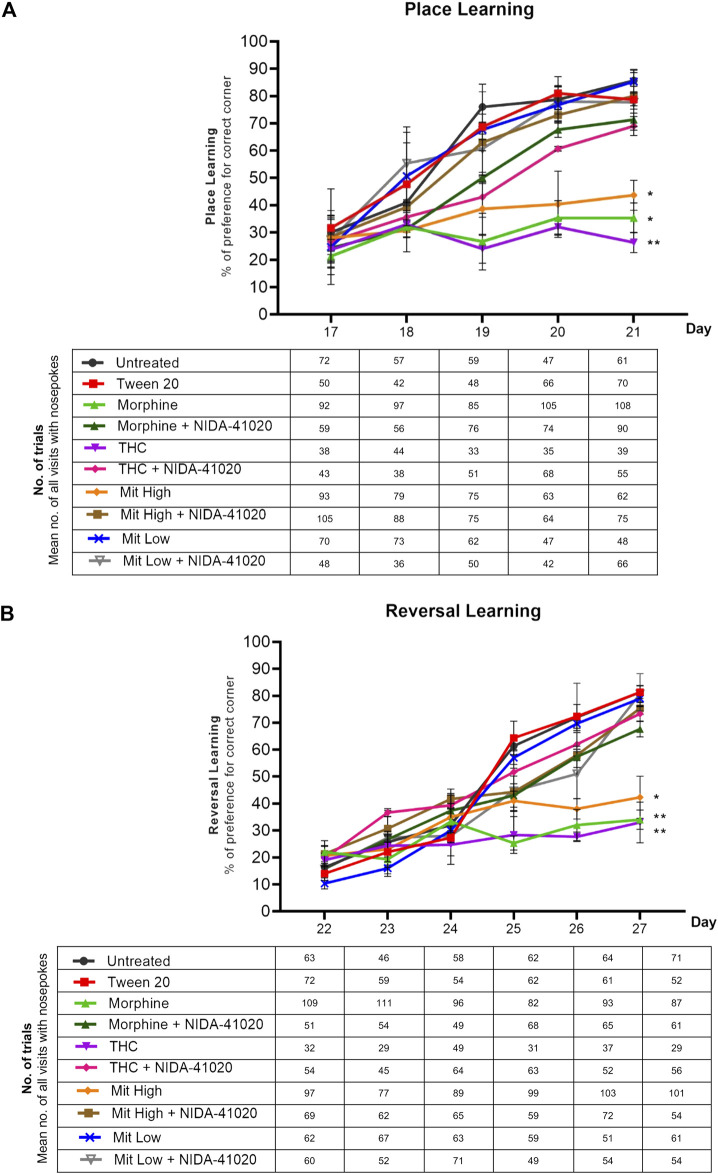
Effects of drug intervention on place learning and reversal learning, and NIDA-41020 antagonism. Data are shown as the percentage of preference for the correct (water-reinforced) corner and number of trials (the mean number of all visits with nose pokes are shown in the table below the graph) **(A)** during the 5 days of place learning phase, as well as **(B)** 6 consecutive days of the reversal learning phase. Data revealed highly significant learning and reversal learning in untreated and Tween 20 control groups and the low-dose mitragynine group. The morphine-, THC-, and high-dose mitragynine-treated groups failed to attain place learning and reversal learning. The cannabinoid CB_1_ receptor antagonist NIDA-41020 significantly reversed morphine-, THC-, and mitragynine-induced place learning and reversal learning impairment in mice. *p < 0.05; **p < 0.01 vs Tween 20 (two-way ANOVA with *post-hoc* Tukey; n = 10/group).

In the 5-day learning phase, two-way repeated measures ANOVA yielded highly significant treatment-by-day place learning in the IntelliCage system (Figure 4A; effect of treatment: F_5, 54_ = 5.571, *p* = 0.023; effect of day: F_4, 216_ = 8.854, *p* = 0.005; treatment × day interaction: F_20, 216_ = 2.659, *p* = 0.014). Untreated, Tween-20 vehicle, and low-dose mitragynine groups showed significant greater visit preference at the correct corner over days (*p* < 0.0001), signifying their improved place learning. However, chronic morphine, THC, and high-dose mitragynine groups showed significant deficits in the acquisition of place learning from Day 17–21 when compared with the control group (morphine: *p* = 0.011, THC: *p* = 0.002, mitragynine: *p* = 0.02). Analyses also discovered that the place learning deficiency in high-dose mitragynine mice did not differ significantly from the morphine- or THC-sensitized mice (*p* = 0.810 vs morphine; *p* = 0.243 vs THC).

Similarly, repeated measures ANOVA conducted on reversal learning data confirmed significant differences in treatment-by-day relearning (Figure 4B; effect of treatment: F_5, 54_ = 17.00, *p* < 0.001; effect of day: F_5, 270_ = 24.88, *p* < 0.001; treatment × day interaction: F_25, 270_ = 6.981, *p* < 0.001). Overall, the percentage of preference for the new correct corner on Day 22 (the first day of reversal learning) was generally lower than that recorded during Day 17 (the first day of learning phase). Significant increase in acquiring the newly placed correct water-reinforced corner was observed in untreated, Tween-20 vehicle, and low-dose mitragynine groups, implying their significant relearning abilities (*p* < 0.05). Consistent with place learning data, all drug-sensitized groups failed to acquire the reversed correct corner (morphine: *p* = 0.004, THC: *p* = 0.002, high-dose mitragynine: *p* = 0.02 vs control). There were no significant differences in reversal learning deficiency observed between high-dose mitragynine-, morphine-, and THC-sensitized groups (high-dose mitragynine: *p* = 0.854 vs morphine; *p* = 0.898 vs THC).

### NIDA-41020 Reverses Mitragynine-Induced Place Learning and Reversal Learning Deficits

A two-way repeated measures ANOVA showed the significant effect of NIDA-41020 on drug-induced place learning (Figure 4A; effect of treatment: F_7, 72_ = 8.246, *p* = 0.0006; effect of day: F_4, 288_ = 70.73, *p* < 0.0001; treatment × day interaction: F_28, 288_ = 5.624, *p* < 0.0001) and reversal learning (Figure 4B; effect of treatment: F_7, 72_ = 15.07, *p* < 0.0001; effect of day: F_5, 360_ = 94.25, *p* < 0.0001; treatment × day interaction: F_35, 360_ = 5.821, *p* < 0.0001). Drugs paired with NIDA-41020 groups showed gradual increased preference for water-reinforced corner, revealing that NIDA-41020 significantly reversed morphine-, THC-, and high-dose mitragynine-induced impairment of place learning (Figure 4A; morphine: *p* = 0.0475 vs morphine + NIDA-41020; THC: *p* = 0.0079 vs THC + NIDA-41020; high-dose mitragynine: *p* = 0.0466 vs high-dose mitragynine + NIDA-41020) and reversal learning (Figure 4B; morphine: *p* = 0.0152 vs morphine + NIDA-41020; THC: *p* = 0.0429 vs THC + NIDA-41020; high-dose mitragynine: *p* = 0.0445 vs high-dose mitragynine + NIDA-41020).

### Immunostaining of Positive and Negative Controls for CB_1_ Receptor Antibody

**FIGURE 5:**
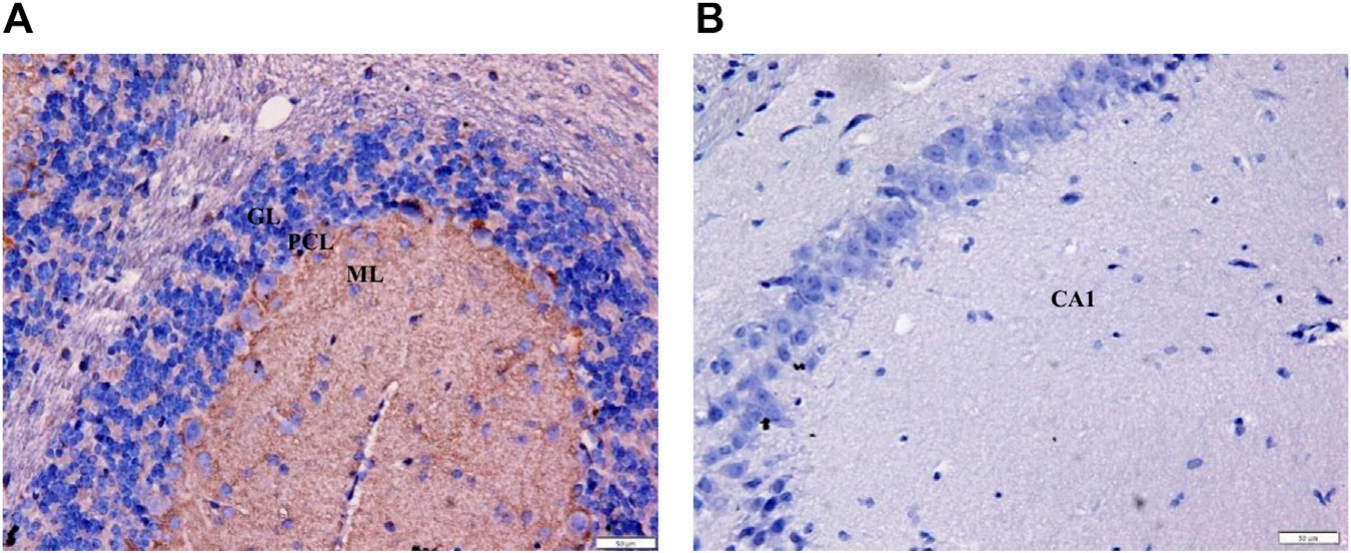
Immunohistochemistry of positive and negative controls at ×400 magnification to validate staining specificity. **(A)** Immunoreactive fibers within the mouse cerebellar molecular layer served as the positive control for CB_1_ receptor. The brown deposits confirm the presence of CB_1_ receptors (OD = 0.28). **(B)** No stain was detected in the neurons and surrounding fibers of the CA1 hippocampal region (negative control; OD = 0.00). GL = granular layer; PCL = Purkinje cell layer; ML = molecular layer; Bars = 50 µm.

The positive immunostaining reaction for mouse cerebellum was presented as brown deposits as seen in the immunoreactive fibers of the molecular layer (positive control; mean OD = 0.28; Figure 5A). Immunostaining of mouse CA1 hippocampal region as a negative control (primary antibodies were omitted) demonstrated the absence of immunostaining in the neurons and the surrounding fibers (negative control; mean OD = 0.00; Figure 5B).

### Mitragynine Increases CB_1_ Receptor Immunoreactivity in the Hippocampal CA1 Region

**FIGURE 6:**
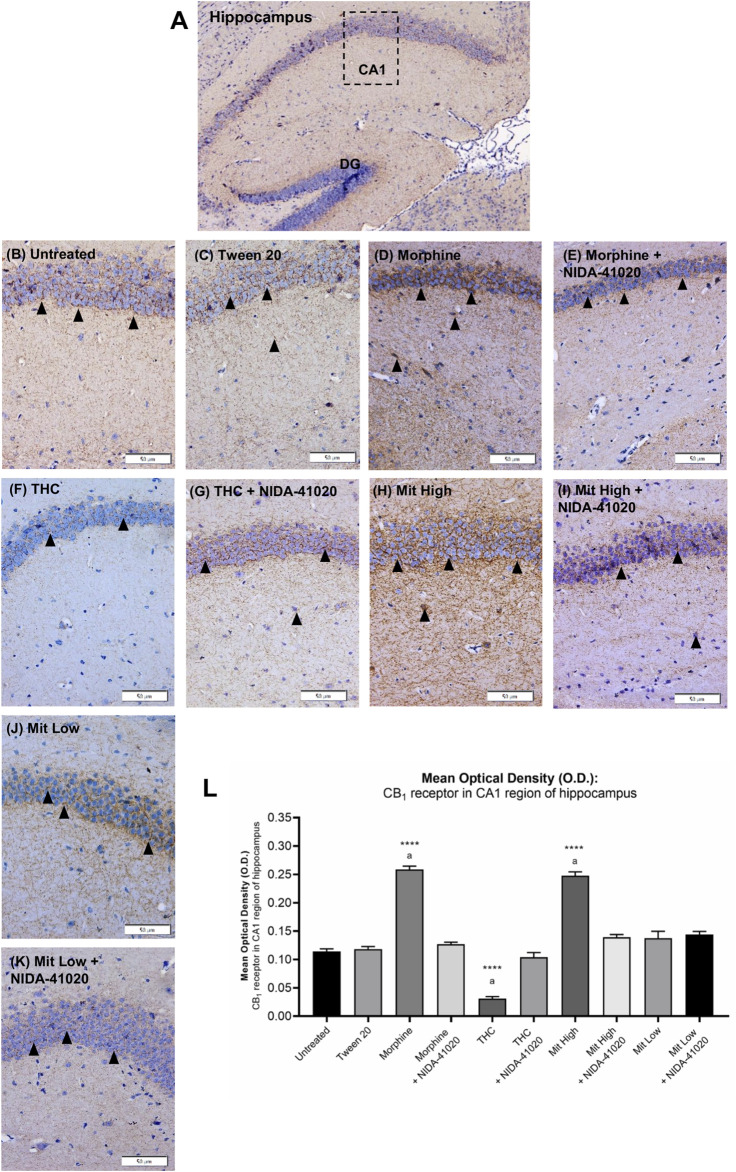
Effects of drug intervention on CB_1_ receptor staining in the **(A)** CA1 pyramidal region of the mice hippocampus (×100 magnification). The micrographs represent **(B)** untreated, **(C)** Tween 20, **(D)** morphine, **(E)** morphine + NIDA-41020, **(F)** THC, **(G)** THC + NIDA-41020, **(H)** high-dose mitragynine, **(I)** high-dose mitragynine + NIDA-41020, **(J)** low-dose mitragynine, and **(K)** low-dose mitragynine + NIDA-41020 groups at ×400 magnification. Arrowheads indicate CA1 pyramidal neurons surrounded by dense plexus of CB_1_ receptor immunoreactive fibers. **(L)** Densitometric analysis are shown as the mean + SEM of 15 replicates per group. ****p < 0.0001 vs Tween 20; a: *p* < 0.05 vs drug + NIDA-41020 groups (one-way ANOVA followed by *post-hoc* Tukey test). Bars = 50 µm.

In the vehicle-treated mice, weak CB_1_ receptor immunoreactivity was observed in CA1 pyramidal neurons surrounded by dense plexus of immunoreactive fibers (Figure 6). There was a statistically significant difference between the groups as determined by one-way ANOVA (Figures 6B–L; F_9, 140_ = 208.7, *p* < 0.0001). Tukey *post-hoc* test revealed that chronic treatment with morphine (Figures 6D,L; *p* < 0.0001) and high-dose mitragynine (Figures 6H,L; *p* < 0.0001), but not low-dose mitragynine (Figures 6J,L; *p* = 0.1622), significantly increased CB_1_ receptor immunoreactivity in the CA1 field in comparison to the control groups. No significant different was detected in the CB_1_ receptor immunoreactivity between morphine-sensitized and high-dose mitragynine-sensitized groups (*p* = 0.8497). By contrast, CB_1_ receptor immunoreactivity in chronic THC was significantly decreased in the CA1 field (Figures 6F,L; *p* = 0.0001 vs control). There was no statistically significant difference in the morphine + NIDA-41020, THC + NIDA-41020, and mitragynine + NIDA-41020 groups compared with the control group (Figures 6E,G,I,L; morphine + NIDA-41020: *p* = 0.7197; THC + NIDA-41020 group: *p* = 0.8868; high-dose mitragynine + NIDA-41020 group: *p* = 0.0364 vs untreated). This suggests a reversal of morphine, THC, and mitragynine effects by CB_1_ receptor antagonism.

### Mitragynine Increases CB_1_ Receptor Immunoreactivity in VTA

**FIGURE 7:**
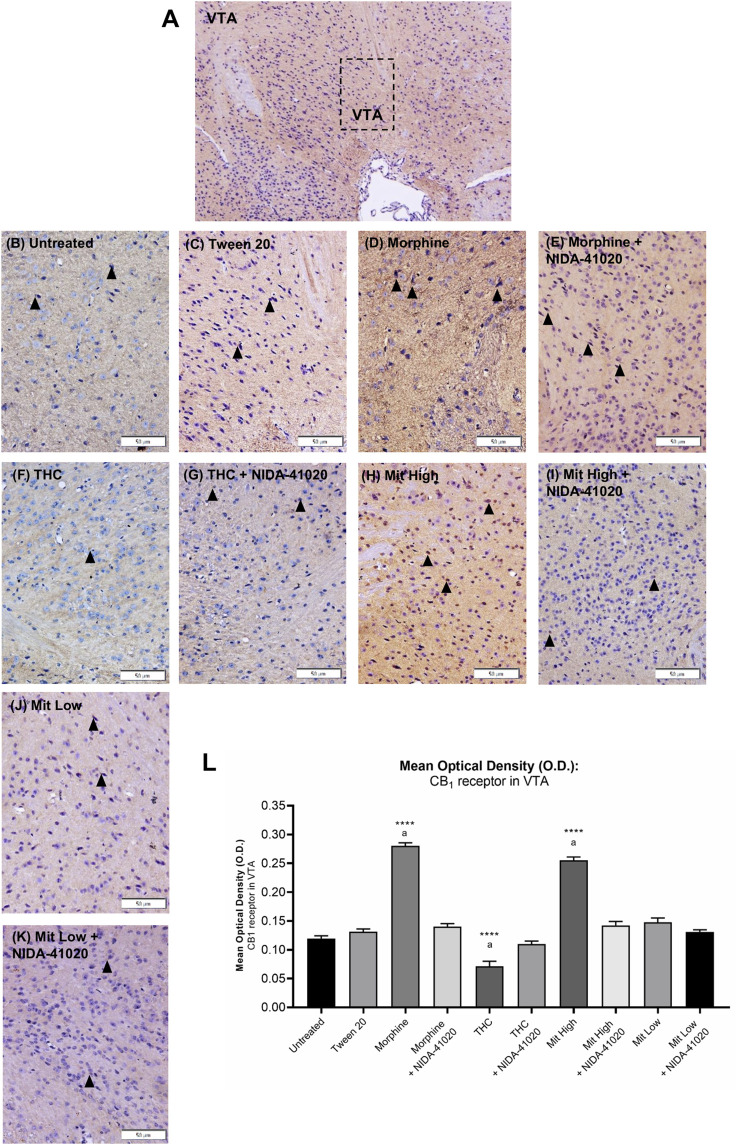
Effects of drug intervention on CB_1_ receptor staining in **(A)** mice VTA (×100 magnification). The micrographs represent **(B)** untreated, **(C)** Tween 20, **(D)** morphine, **(E)** morphine + NIDA-41020, **(F)** THC, **(G)** THC + NIDA-41020, **(H)** high-dose mitragynine, **(I)** high-dose mitragynine + NIDA-41020, **(J)** low-dose mitragynine, and **(K)** low-dose mitragynine + NIDA-41020 groups at ×400 magnification. Arrowheads indicate CB_1_ immunoreactive neurons and the surrounding fibers. **(L)** Densitometric analysis are shown as mean + SEM of 15 replicates per group. ****p < 0.0001 vs Tween 20; a: *p* < 0.05 vs drug + NIDA-41020 groups (one-way ANOVA followed by *post-hoc* Tukey test). Bars = 50 µm.

In the VTA, moderate CB_1_ receptor immunoreactivity was observed in neuronal cell bodies and the surrounding fibers of vehicle-treated mice (Figure 7). A one-way ANOVA showed statistically significant difference between the groups (Figures 7B–L; F_9, 140_ = 106.2, *p* < 0.0001). Chronic morphine (Figures 7D,L; *p* < 0.0001) and high-dose mitragynine (Figures 7H,L; *p* < 0.0001) groups, but not low-dose mitragynine (Figures 7J,L; *p* = 0.087), showed significant increment of CB_1_ receptor immunoreactivity in the VTA compared to the Tween 20 vehicle group. There was no significant difference between the morphine- and mitragynine-sensitized groups (*p* = 0.0624). By contrast, CB_1_ receptor immunoreactivity in chronic THC was significantly decreased in the VTA (Figures 7F,L; *p* < 0.0001 vs vehicle). CB_1_ receptor immunoreactivity in the VTA of morphine + NIDA-41020, THC + NIDA-41020, and mitragynine + NIDA-41020 groups did not show any significant difference compared with the Tween 20 group (Figures 7E,G,I,L; morphine + NIDA-41020: *p* = 0.2282; THC + NIDA-41020 group: *p* = 0.9499; high-dose mitragynine + NIDA-41020 group: *p* = 0.1349 vs vehicle), which suggests a reversal of the morphine, THC, and mitragynine effects.

### Mitragynine-Induced Upregulation of CB_1_ Receptor Levels in Brain Reward Area Reversed by CB_1_ Receptor Antagonism

**FIGURE 8:**
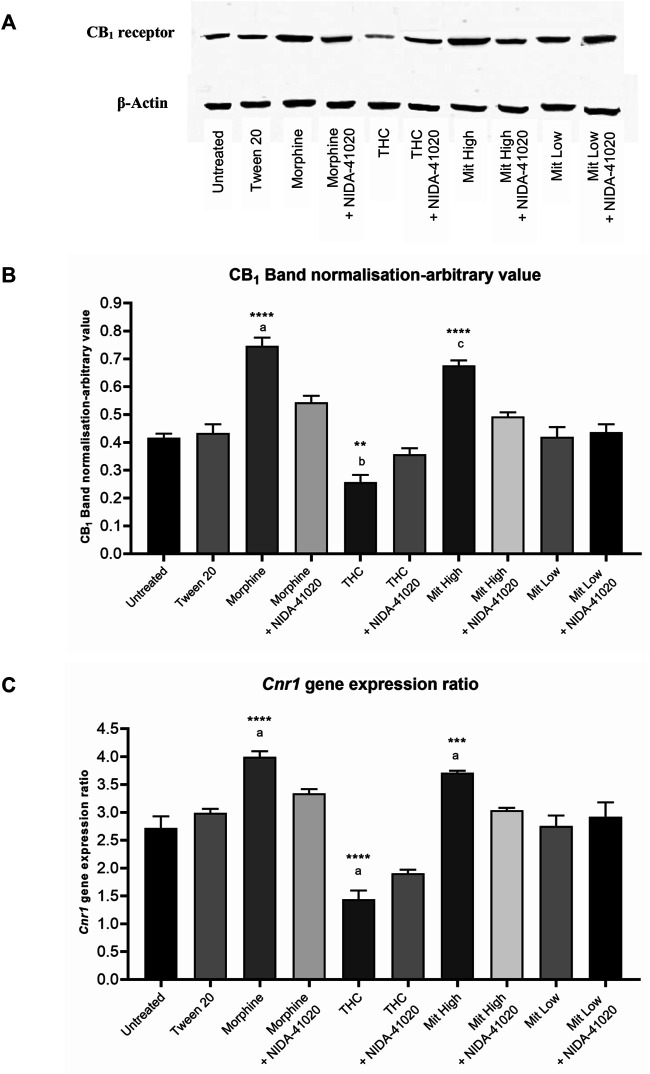
CB_1_ receptor **(A, B)** protein and **(C)** gene levels in mice brain mesolimbic area after 28 days of drug intervention. Data are shown as the mean + SEM of three biological replicates per group. The CB_1_ receptor values have been normalized to β-actin controls. Data revealed that high-dose mitragynine and morphine groups significantly increased, whereas THC decreased; CB_1_ receptor expression in the brain mesolimbic area vs controls and low-dose mitragynine group. NIDA-41020 reversed the drug-induced CB_1_ receptor alteration. ***p < 0.001, ****p < 0.0001 vs Tween 20; a: *p* < 0.05 vs drug + NIDA-41020 groups (one-way ANOVA followed by *post-hoc* Tukey test).

The CB_1_ receptor protein levels in the brain reward mesolimbic area, as assessed by western blot, showed an overall significant difference between the groups (Figures 8A,B; F_9, 20_ = 50.16, *p* < 0.0001). The western blot analysis displayed that the protein levels of CB_1_ receptor were upregulated in the morphine-sensitized (*p* < 0.0001) and high-dose mitragynine-sensitized groups (*p* < 0.0001), whereas downregulated in the THC-sensitized group (*p* = 0.0022), when compared with that in the vehicle group. There were no significant differences between the morphine- and mitragynine-sensitized groups (*p* = 0.437). No significant differences in CB_1_ receptor protein levels of low-dose mitragynine were detected (*p* = 0.712). The administration of NIDA-41020 significantly reversed the drug-induced alterations of CB_1_ receptor protein expressions as demonstrated in the respective drug + NIDA-41020 groups which do not differ significantly from the vehicle group (morphine + NIDA-41020 group: *p* = 0.0734; THC + NIDA-41020 group: *p* = 0.3405; high-dose mitragynine + NIDA-41020 group: *p* = 0.6012 vs vehicle).

The qPCR analysis was further performed to evaluate the effect of chronic drugs, with/without NIDA-41020 coadministration, on the CB_1_ receptor at the mRNA level in the brain mesolimbic area (Figure 8C; F_9, 20_ = 113.6, *p* < 0.0001). Consistent with the western blot results, the qPCR analysis also showed that chronic morphine (*p* < 0.0001) and mitragynine (*p* = 0.0006) triggered the upregulation, while chronic THC triggered the downregulation (*p* < 0.0001), of the *Cnr1* gene level compared to vehicle. No significant differences were observed between the morphine- and mitragynine-sensitized groups (*p* = 0.3225). No significant difference was also observed in the low-dose mitragynine group compared with the control (*p* = 0.251). The drug-induced alteration of the *Cnr1* gene levels appeared to be absent in the respective drug + NIDA-41020 groups (morphine + NIDA-41020 group: *p* = 0.1358; THC + NIDA-41020 group: *p* = 0.0568; high-dose mitragynine + NIDA-41020 group: *p* = 0.9995 vs vehicle). These findings further affirm the likely CB_1_ receptor antagonism of morphine, THC, and high-dose mitragynine at the protein and gene levels.

## Discussion

The primary goal of the present study was to identify the involvement of cannabinoid CB_1_ receptors in mediating the behavioral and cognitive effects of chronic kratom/mitragynine exposure. The IntelliCage system has been validated and widely used for addiction-related mouse models for various substances (Radwanska and Kaczmarek, 2012; Iman et al., 2017; Skupio et al., 2017; Ajonijebu et al., 2018; 2019). In this study, the IntelliCage system was adapted for novelty-seeking trait (exploration of a novel environment) and basal horizontal locomotor activity levels in a familiar environment, in order to characterize the behavioral exploratory patterns (i.e., measured by the number of corner visits) in group-housed Swiss albino mice before drug intervention. During the initial adaptation period in the IntelliCage, all mice from all groups demonstrated similar pre-intervention novelty-seeking exploration and baseline locomotor activities shown by the number of visits to all corners. However, slight insignificant differences recorded between the groups may have originated because of individual differences or variations in the mice. Individual variations are often caused by the physical and social environment during development and adult life. This may be a key factor for the weak reproducibility of animal experiments (Koolhaas et al., 2010; Toth, 2015; Müller, 2018). Individual variations in drug vulnerability that may arise from poorly standardized housing, experimental protocols, and experimenter's handling, as well as social isolation, will jeopardize the reliability. To minimize these problems, an automated social group instrument such as the IntelliCage system was used in this study, as had been previously documented in addiction-related research (Radwanska and Kaczmarek, 2012; Marut et al., 2017).

This study revealed that chronic high-dose mitragynine (5–25 mg/kg) significantly enhanced exploratory activity in mice, as shown by the increased number of IntelliCage corner visits. Evidently, acute mitragynine administration was reported to induce a significant increase of arm explorations in Y-maze and elevated plus maze tests, as well as central zone explorations in the open-field test (Hazim et al., 2011; Hazim et al., 2014; Yusoff et al., 2016). Studies have suggested that in response to repeated administration of abused substances, locomotor sensitization occurs, resulting in the progressive amplification of behavioral and locomotor activity (Koob and Volkow, 2016; Uhl et al., 2019). Similarly, an acute low dose of mitragynine (1 mg/kg) induced profound hyperlocomotion and rearing activities in the open-field task (Yusoff et al., 2016). However, this finding contrasts with the findings of Apryani et al. (2010) that reported a significant reduction in locomotor activity in the open-field test after 28 days of mitragynine administrations (5, 10, and 15 mg/kg). The behavioral differences may reflect the psychostimulant effects with earlier exposures to mitragynine (i.e., within 7 days of administration) in this study. Subsequent behavioral and neural adaptations following chronic use may ultimately result in motor deficit. Despite the reported hypo- and hyperlocomotion induced by mitragynine at various treatment regimes and doses, the chronic low-dose mitragynine (1–4 mg/kg) in this study exhibited unaltered locomotor activity shown by a similar number of IntelliCage corner visits compared with the untreated and vehicle groups. This is in agreement with the previous findings of Moklas et al. (2008) that found unaltered locomotion activity when using 1 mg/kg mitragynine in a locomotor box, but is in contrast to the findings of Yusoff et al. (2016). Overall, this signifies the lack or no effect of chronic low-dose mitragynine on locomotor sensitization, unlike the effect induced by high-dose mitragynine, morphine, and THC.

Morphine-treated mice exhibit hyperlocomotion, which coincides with the induction of progressive behavioral sensitization in mice with intermittent morphine administration (Li et al., 2010; Kitanaka et al., 2018). By contrast, repeated exposure to THC produced a decrease in the number of visits to IntelliCage corners, signifying reduced locomotor activity in mice, which is consistent with several previous studies examining the locomotor effects of THC (Schramm-Sapyta et al., 2007; Dow-Edwards and Izenwasser, 2012; Bergman et al., 2016). Repetitive THC treatment in human and animal studies induced behavioral tolerance, which coincided with a rapid downregulation and desensitization of cannabinoid receptor–binding sites in several brain areas of the mesocorticolimbic circuitry and cerebellum (Justinova et al., 2009; Burston et al., 2010). This neural adaptation seems to be responsible for the development of cannabinoid tolerance, causing subsequently diminished locomotion. Furthermore, the anxiogenic effects of acute and chronic THC may contribute to hippocampal GABAergic dysfunction (Schramm-Sapyta et al., 2007), thereby exacerbating locomotor impairments.

In this study, the mice those were chronically treated with high-dose mitragynine (5–25 mg/kg), morphine, and THC showed significantly higher preferences for the sucrose-associated corner than did those untreated, vehicle treated, and treated with low-dose mitragynine (1–4 mg/kg). These findings suggest the escalation of sucrose hedonic value in drug-treated mice. Sweet preference has been speculated to be a predictor of substance abuse because of similar overlapping anatomical and functional mechanisms related with the rewarding effects of addictive substances and sucrose (Smith et al., 2011; Nieh et al., 2015). It is also feasible that the behavioral and neurological changes accompanying substance dependence result in enhanced impulsivity to securing immediate natural rewards (O'Brien et al., 2013; Harvey-Lewis et al., 2015). Contradictory to the increase of sucrose hedonic value by high-dose mitragynine sensitization, low-dose mitragynine did not induce any significant alteration in sucrose preference. This finding is in parallel with the findings of Yusoff et al. (2016) that supported the dose-dependent effects of mitragynine on its rewarding properties, where mitragynine at 1 and 5 mg/kg was found to suppress condition-placed preference (CPP) when compared with 10 and 30 mg/kg of mitragynine that induced CPP similar to 10 mg/kg morphine.

In addition, the present study also showed the consolidation of aversive memory to air-puff punishment as evident by the decreased persistency in sucrose seeking with a decrease in the frequency of licks at the sucrose-associated corner when paired with air-puff punishment. Liu et al. (2014) described a significant increase in mice heart rate in response to sudden air-puff stimuli, suggesting activation of a fearful emotional state. A fearful event leads to the alteration of the hippocampal CA1 region and anterior cingulate cortex, thus evoking aversive memory to that event (Xie et al., 2013). This may imply the possibility of enhanced resistance to air-puff punishment, as well as heightened persistency and motivation in securing salient reward in drug-dependent mice. Neuroplasticity in the mesolimbic dopaminergic system, especially in the amygdala that governs emotion and fear conditioning, may result in making sensitized mice exhibit an enhanced ability to resist punishment. Additionally, the extinction of long-lasting fear and aversive memory may occur with repeated exposure to addictive drugs. This is consistent with the literature on the impaired memory processes evoked by acute and chronic treatment with mitragynine (Yusoff et al., 2016; Iman et al., 2017), morphine (Lu et al., 2010), and THC (Justinova et al., 2009; Calabrese and Rubio-Casillas, 2018). Studies have also demonstrated that rodents were willing to endure punishment in pursuit of abused drugs (Vanderschuren et al., 2017), which appears to mimic the pathological loss of control over substance abuse (or compulsive behaviors) seen in human addicts. Altogether, this compulsivity points to the underlying motivational shift from substance “liking” to “wanting” phenomenon even when the substance is no longer pleasurable. Nonetheless, mice from the high-dose mitragynine group showed significantly higher persistency in obtaining sucrose than those in the low-dose mitragynine group, which may indicate the dose-dependent effect on sucrose persistency that denotes dose-dependent compulsivity induced by mitragynine.

The place learning and reversal learning protocols were used to evaluate the effects of mitragynine sensitization on the learning abilities, as compared with the effects of morphine and THC. Learning abilities were determined by the percentage of visits (with successive nose pokes) performed at the water-reinforced corner. Untreated, Tween 20, and low-dose mitragynine groups showed place learning and reversal learning. The finding from the low-dose mitragynine group is in accordance with the findings of a study by Hassan et al. (2019) in which low-dose mitragynine (1 mg/kg)–treated mice showed preserved learning abilities in the Morris water maze task. In this study, we found that high-dose mitragynine-, morphine-, and THC-sensitized mice failed to learn the location of the water-reinforced corner. All mice from the three drug-sensitized groups showed no place learning and failed to show efficient reversal learning. Cognitive and learning deficits had been demonstrated in several studies after acute and chronic mitragynine treatment (Apryani et al., 2010; Hazim et al., 2011; Yusoff et al., 2016; Iman et al., 2017; Hassan et al., 2019). Recent human cross-sectional studies report progressive dependency, tolerance, craving, and withdrawal symptoms during abstinence from kratom consumption. These studies also suggest an association of poor cognitive performance with chronic kratom use in humans (Vicknasingam et al., 2010; Saingam et al., 2013; Cinosi et al., 2015; Singh et al., 2019). The long-lasting cognitive changes may eventually disrupt inhibitory control and impair decision-making, thereby contributing to a loss of control over drug intake, which reflects the persistent pursuits of drugs seen in human addicts. Additionally, cognitive deficits in the realm of learning and memory that persist even after prolonged abstinence may also impede the success of rehabilitation programs and thus provoke subsequent relapse despite achieving remission.

Reward-related place learning is a hippocampal-dependent task, paralleled by concomitant changes in VTA function and functional connectivity (Gomperts et al., 2015; Gruber et al., 2016; Pytka et al., 2020). Therefore, neuroplasticity in the hippocampus and VTA following chronic exposure to addictive substances may account for place and reversal learning impairment. This is consistent with reports of place learning deficits accompanied by reduced CA1 hippocampal synaptic transmission and LTP following high-dose mitragynine in rats (Hassan et al., 2019). CA1 hippocampal dysfunctions were also observed in rodents treated with a methanolic kratom extract (Harizal et al., 2012), morphine (Yang et al., 2013), and THC (Laaris et al., 2010). However, the causality remains unclear.

The CB_1_ receptor antagonist NIDA-41020 was administered to mitragynine-sensitized mice to investigate the involvement of the endocannabinoid system in mitragynine-induced cognitive impairments. The selection of NIDA-41020 as the CB_1_ receptor antagonist reaffirmed CB_1_ receptor modulation that are known to occur with CB_1_ receptor antagonism/reversal effect on morphine and THC (Pickel et al., 2004; Jin et al., 2014; Schindler et al., 2016). Behavioral alterations in both morphine and THC groups in the presence of NIDA-41020 when compared with the mitragynine groups became the surrogates for the likely CB_1_ receptor involvement. Hence, NIDA-41020–alone group was not included in the present study. Interestingly, upon administration of NIDA-41020, mice treated with high-dose mitragynine showed a progressive ability in learning the location of the water-reinforced corner. This finding demonstrates that NIDA-41020 reversed the learning impairment in mitragynine-sensitized mice, thus providing the first clue to the interaction of mitragynine with the endocannabinoid system. In fact, this occurs during abstinence (i.e., 14 days without injection of drugs), and the reversal effect is comparable to the reversal effect produced by NIDA-41020 in the morphine- and THC-treated groups, suggesting that chronic high-dose mitragynine may exert its effect on CB_1_ receptor signaling in probably the same way as would morphine and THC. It has been shown that long-term learning and memory impairment and the underlying neuronal alterations persist even after abstinence, particularly in the case of chronic administration of morphine, THC, and high-dose mitragynine (>5 mg/kg in mice) (Jin et al., 2014; Schindler et al., 2016; Iman et al., 2017; Hassan et al., 2019; Valentinova et al., 2019). Although this was adopted in the present study design, a similar interpretation cannot be made on the low-dose mitragynine (1–4 mg/kg) and limited by the absence of the NIDA-41020-alone group. Nevertheless, this gradual improvement in place learning ability with repetitive use of NIDA-41020 may suggest the prospective use of CB_1_ receptor antagonists presumably by mitigating the effect of substance-induced learning memory deficits in chronic kratom users, and as a potential treatment for substance use disorder. The findings of this study also lend support to previous studies demonstrating that NIDA-41020 administration blocked THC, ethanol, and nicotine self-administration and reinstated substance-seeking behaviors in rodents (de Bruin et al., 2011; Maldonado et al., 2013; Schindler et al., 2016).

Previous molecular and histology studies showed that in the mesocorticolimbic reward pathway, a significantly high density of CB_1_ receptor protein was localized on both GABAergic and glutamatergic axon terminals of the hippocampus, whereas a modest CB_1_ receptor localization was reported throughout the neocortex, VTA, amygdala, periaqueductal gray nucleus, nucleus accumbens, and medial hypothalamus (Tsou et al., 1998; Katona, 2009). In the present study, the quantitative analysis of CB_1_ expression focused on the hippocampal CA1 region and VTA. Tsou et al. (1998) showed that CB_1_ receptors were expressed at the lightly stained cell bodies of CA1 pyramidal neurons, surrounded by a dense plexus of immunoreactive fibers. In the VTA, lightly stained CB_1_ immunoreactive neurons and the surrounding fibers were located on the floor of the midbrain, adjacent to the substantia nigra (Tsou et al., 1998). The present study revealed an upregulation of CB_1_ immunoreactive fibers within the CA1 pyramidal region of the hippocampus, as well as CB_1_ immunoreactive neurons and fibers in the VTA of mitragynine-sensitized mice. Consistently, the western blot and qPCR protocols also demonstrated a significant upregulation of CB_1_ receptor protein and mRNA levels in these mice. CB_1_ receptor protein upregulation may be a biochemical marker related to the development of tolerance and dependence after chronic mitragynine treatment. These upregulations suggest that adaptations within the endocannabinoid system may account for the observed learning impairments. The mitragynine-induced CB_1_ receptor upregulations appeared to be attenuated by NIDA-41020 as seen in the immunohistochemical, protein, and mRNA studies. These findings suggest a potential mechanism for the beneficial effects of CB_1_ receptor antagonism at the behavioral level. Therefore, the present results could also indicate a plausible chronic mitragynine–CB_1_ receptor interaction in inducing the transition from “liking” to “wanting” response, which eventually culminates in the development of mitragynine/kratom addiction (Nanthini et al., 2015). Similarly, morphine-treated mice were also found to show an upregulation of CB_1_ receptors in the hippocampal CA1 region and VTA at the immunohistochemical as well as protein and mRNA levels. Correspondingly, a study by Jin et al. (2014) demonstrated the upregulation of brain CB_1_ receptor protein and mRNA levels occurring in morphine-dependent mice. In this study, we found that morphine effects were also reversed by CB_1_ receptor antagonist treatment which confirms previous findings by Yamaguchi et al. (2001), extending the potential of CB_1_ receptor antagonism as a treatment strategy to mitigate cognitive deficits in opiate addicts.

By contrast, THC-sensitized mice produced a significant downregulation of CB_1_ receptor at the protein and mRNA levels in the CA1 hippocampal region and VTA. These findings are consistent with previous reports demonstrating the significant decline of CB_1_ binding sites in several brain areas, including the mesolimbic system following chronic administration of cannabinoids in animals (Justinova et al., 2009) and humans (Hirvonen et al., 2012). Persistent abuse of marijuana or THC seems to be responsible for the development of profound cannabinoid tolerance which correlates with desensitization and downregulation of CB_1_ receptors. Conversely, mice treated with chronic low-dose mitragynine exhibited unaltered regulation of CB_1_ immunoreactivity within the hippocampal CA1 region and VTA, along with the unaltered CB_1_ receptor protein and gene expressions, such as in the control group. These findings support the view of little or no endocannabinoid adaptations after chronic low-dose mitragynine.

## Conclusion

The data of the present study demonstrate that high-dose mitragynine can induce spatial/place learning deficits in mice that resemble those of morphine and THC. This was paralleled by an induction of morphine-like CB_1_ receptor alterations in mitragynine-sensitized mice, reaffirming the likelihood of common hijacking of the related brain pathways. This substantiates the kratom/mitragynine risk of abuse, dependence, and addiction after prolonged and unregulated use. The CB_1_ receptor antagonist, NIDA-41020, reversed the behavioral and neural changes associated with prolonged mitragynine exposure. Furthermore, future research on binding dynamics, molecular docking studies of the CB_1_ and CB_1_-medicated signaling, and NIDA-41020–alone group in the experimental design may further substantiate CB_1_ receptor involvement in kratom/mitragynine addiction. In conclusion, findings from the present study are the first to suggest a plausible role of CB_1_ receptor in mediating the dose-dependent cognitive impairments after chronic high-dose mitragynine. This also highlights the potential of CB_1_ receptor antagonism in ameliorating the cognitive deficits associated with long-term kratom/mitragynine consumption in humans.

## Data Availability

The data sets presented in this study can be found in online repositories. The names of the repository/repositories and accession number(s) can be found in the article/supplementary material.
